# Effect of Presowing Treatments on Seed Germination and Growth Performance of 1-Year *Lagerstroemia speciosa* (L.) Pers Seedlings in Northeast India

**DOI:** 10.1155/tswj/8127830

**Published:** 2025-05-05

**Authors:** Faisal Ismail Musa, Uttam Kumar Sahoo

**Affiliations:** ^1^Department of Forestry, University of Blue Nile, Ad-Damzin, Sudan; ^2^Department of Forestry, Mizoram University, Aizawl, Mizoram, India

**Keywords:** *Lagerstroemia speciosa*, seedling growth performance, seed presowing treatments

## Abstract

*Lagerstroemia speciosa* (L.) Pers (Lythraceae family), called pride of India, is an important ornamental and medicinal plant having a very high demand for its fruits. Six presowing treatments, that is, hot water, cold treatment, cow dung, H_2_SO_4_, gibberellic acid (GA_3_), and scarification, were used to evaluate the effect of treatments on seed germination, mean germination time, and germination energy and monitor the growth performance of seedling as well as seedling quality index of *L. speciosa*. Among the different treatments, hot water recorded the highest germination percentage (51.95%). However, minimum mean germination time was achieved by H_2_SO_4_ treatment (21.17 days). Additionally, a significant difference (*p* < 0.05) was observed in germination rate among treatments and the number of seeds germinated per day among treatments. The mean seedling height of *L. speciosa* reached a maximum height of 104 cm and a diameter of 10.67 mm in 1 year. We found a strong relation between seedling height and soil temperature (*r* = 0.56) and between seedling characteristics and biomass parameters, while there was a negative correlation with the total percentage of water content. The Dickson quality index of the seedlings was found to be 2.68. Our findings recommend hot water as the best treatment for ensuring quality seedlings, and seedlings of 1 year is sufficiently robust for large-scale afforestation and reforestation programs.

## 1. Introduction

Seed production is an important stage in plant natural regeneration, although it is influenced by a variety of factors including resource availability, failure of pollination, predation on flowers, fruit, tree size and age, genetic constitution, and climatic condition [1]. Seeds are central to the growth cycle in plant [2], which plays an important role in feature of plant regeneration [3, 4] and conservation of plant biodiversity [5]. Several seeds have particular morphological adaptations that influence the movement of seed into suitable germination microsites [6]. However, large seeded species are more tolerant from the shade and germination compared to small seed [7], and seed size has an important consequence for seed germination and survival of plant fitness [8].

Seed germination performance is an important determinant of successful plant regeneration [5, 9]. High-vigour seeds, which exhibit rapid and resilient germination synchronous to environmental stresses, establish healthy seedlings [5]. However, several parental environmental factors influenced seed vigour during seed development and during harvest time as well as storage conditions [10]. Therefore, a decrease in seed quality could lead to a decline in rapidity and germination uniformity [5].

Seed pretreatment can ensure both success in germination speed and an enhanced germination percentage (GP) [11]. Likewise, successful seed germination may increase by adapting appropriate pretreatment techniques [12]. So, in order to produce a large quantity of quality seedlings with lower cost and labor, it is necessary to determine the variation in pretreatments of seeds. Several studies have highlighted the use of pretreatments and confirmed that these can improve the GP, reduce germination time, and produce uniform seedlings suitable for plantation programs [11, 13, 14]. However, the most common treatments reported in literature include hot water, cold treatment, scarification, H_2_SO_4_ with different concentration, cow dung, and gibberellic acid (GA_3_) [12, 15].

**Figure 1:**
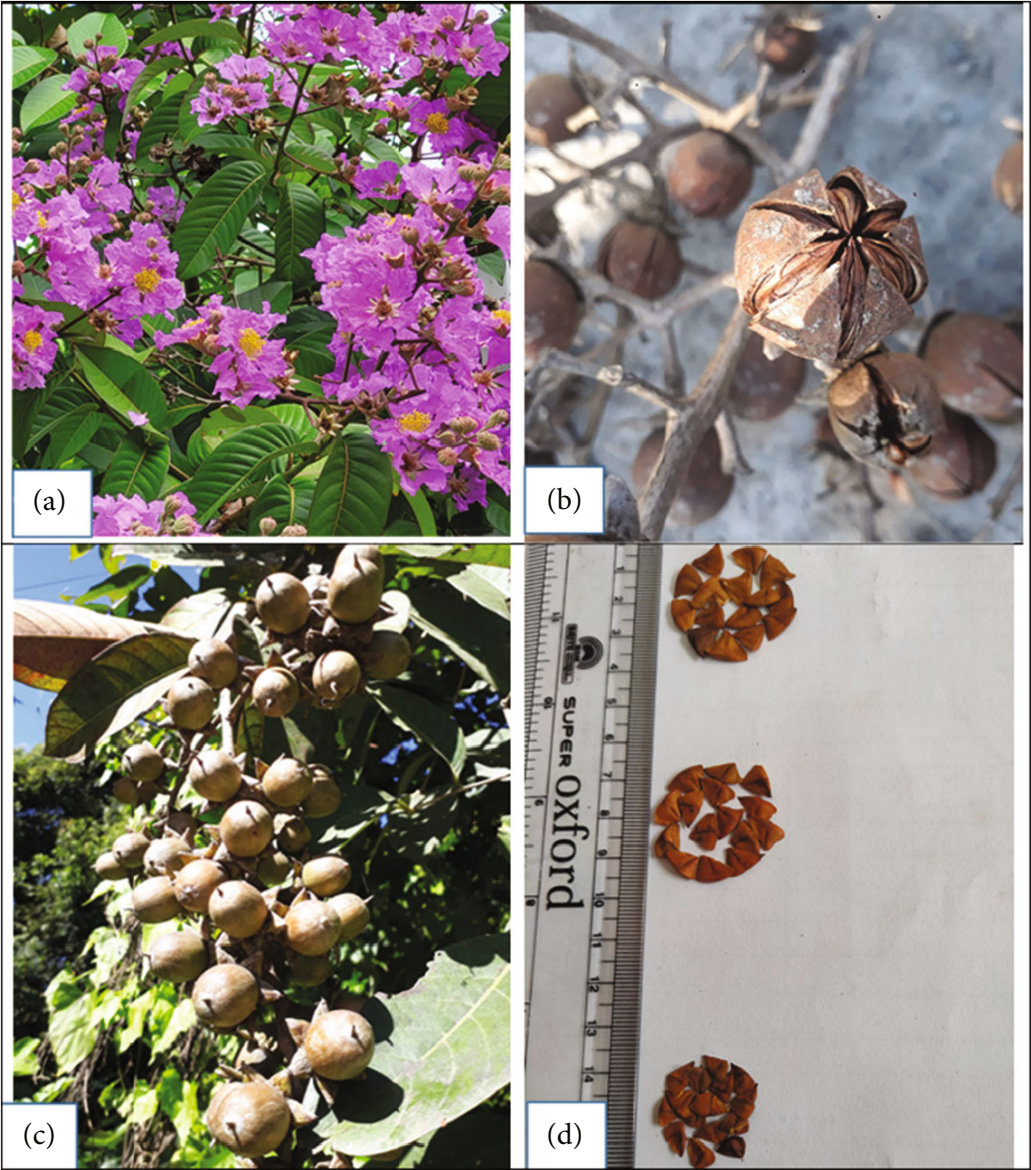
(a) Flowers, (b) fruit capsule, (c) fruits before maturation, and (d) seed of *L. speciosa.*

*Lagerstroemia speciosa* (L.) Pers (Lythraceae family), called pride of India in India [16] and banaba in Philippines [17], is a small to medium-sized tree [18, 19] that grows up to 25 m and 60 cm in diameter [16, 20, 21], with pink or purple flowers (Figure 1a) and capsule fruits (Figure 1b). It is an important ornamental and medicinal tree [22–24] with multipurpose use, highly valued for varying shades of blooms and its medicinal properties in treating diabetes, kidney, obesity, and related ailments [17, 22]. Its bark is used to remove heavy metals such as hexavalent chromium [Cr (VI)] from water [25]. Additionally, the leaves, stem, seeds and bark contain several bioactive compounds that are added to food or food products for enhancement of their health-promoting properties [16, 23]. Its timber is used to make agricultural implements, decorative furniture, pulp, paper, fence posts, ploughs, and boats [16]; besides, waste biomass seed coat of the species is used for manufacturing of biofertilizer [26].

The species is extensively used for afforestation and ecological restoration [27] and in agroforestry [28] due to its dense and wide spreading root systems, diverse traditional applications from medicinal purposes to production of dyes, and flavouring and culinary ingredients [18, 22]. Because of its several horticultural and therapeutic properties, the species has been highly exploited in the natural habitat, resulting in mature tree scarcity [22]. Though the species is insect/cross-pollinated, it has the ability to self-cross under pollen limitation. The self-pollinated seeds are mostly aborted seeds of shorter seed viability having low success rate resulting in low plant density [29]. As the vegetative propagation techniques for this species are not widely practiced [16, 22] and the seeds have shorter viability, it is often difficult to maintain sufficient quality planting stock [30]. Many scientists have reported the low germination rate of *L. speciosa* [31–33] and its low rate of recruitment in natural population [29]. For example, Babele and Kandya [34] reported that only 25% of the newly harvested seeds of *L. speciosa* are viable. The massive and asynchronous pollination in the species leads to a majority of seeds with underdeveloped or no embryo, therefore necessitating optimal seed germination for the viable seeds needed for large-scale plantations. Pretreatment can help reduce germination time and produce good quality of seed and uniform seedling, which can help in all plantation programs of species. Several studies conducted on *L. speciosa* focus on its photosynthetic parameters and oxidative stress [16], bioactive compound [21, 23], medicinal value [17], and micropropagation [22, 35], while there are very limited studies on seed pretreatments.

The presowing treatments of this study were selected based on the physical and physiological dormancy characteristics of *L. speciosa* seeds. Given that species within the *Lagerstroemia* genus often exhibits seed dormancy [12], treatments such as scarification and H_2_SO_4_ were included to improve water permeability and uptake. Additionally, GA_3_ was chosen to assess its role in breaking the physiological dormancy; meanwhile, hot and cold treatments were tested based on their effectiveness in other related tropical tree species. However, cow dung treatment was included as it is a traditional and eco-friendly method used for seed germination improvement in various species. Therefore, this study aims to assess the effect of presowing treatments on germination of *L. speciosa* seeds and identify the best treatment for quality seedling production. Additionally, the study aims to evaluate the growth performance of the seedlings in the greenhouse for 1 year. To achieve this objective, the following questions were addressed: (A) What is the most effective treatment in improving seed germination of *L. speciosa*? (B) What are the minimum and maximum days that seeds take to germinate? (C) What are the maximum seedling height and diameter growth in 1 year? and (D) How does the soil temperature affect seedling height and diameter growth?.

## 2. Method

### 2.1. Seed Germination

**Figure 2:**
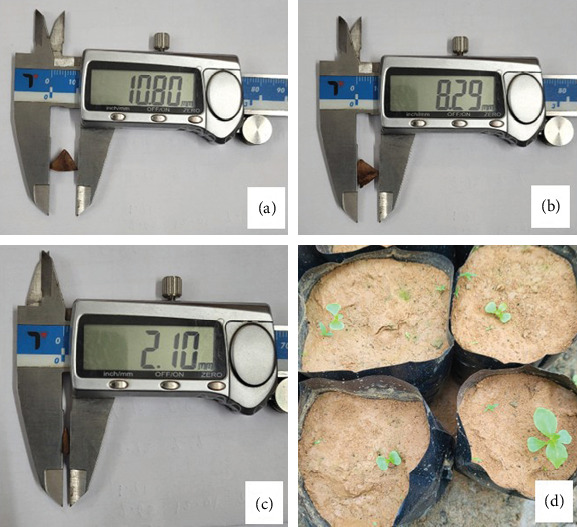
(a–c) *L. speciosa* seed dimension and (d) seed germination at first stage.

The seeds of *L. speciosa* were collected from mature, healthy mother trees in February 2023 from the Mizoram University campus. The seeds were air-dried and separated from the fruit capsule manually. These seeds were stored at room temperature for 1 month before sowing so that they receive similar conditions with the pretreated seeds. The average seed weight was 0.013 ± 0.002 g, while length, breadth, and thickness were 9.15 ± 1.76 mm, 7.36 ± 1.15 mm, and 1.85 ± 0.22 mm, respectively (Figures 2a, 2b, and 2c). The seeds were thoroughly checked, and the discolored and damaged seeds were removed prior to their storage at room temperature. Different pretreatments were used, that is, hot water, cold treatment, cow dung, H_2_SO_4_, GA_3_, scarification, and control. In hot water (80°C) treatments, seeds were soaked for 10 min [12] and 5 min in H_2_SO_4_ (60%). Additionally, the seeds were soaked in GA_3_ solution for 24 h as recommended by [15] and then subjected to cold treatment in a fridge for 24 h. Seeds were grown in polybags where soil was the medium for all treatments. For each treatment, 36 seeds were sown, germination was monitored daily, and the number of seeds germinated was recorded on a daily basis. Additionally, the irrigation was conducted every day to maintain the soil temperature and moisture until the end of the experiment, while the average soil temperature during germination test was 25.45°C, and the soil moisture was rated 8 out of 10. A multithermometer (St-9283) for measuring soil temperature and a FreshDcart (FDC-032) for measuring soil moisture were used.

### 2.2. Seedling Growth Performance in Greenhouse

To assess the growth performance of the seedlings, 10 random seedlings were selected and carefully transplanted in large poly-pots (30 cm × 45 cm). The experiment was conducted in a greenhouse where temperature was 25°C–35°C and 15°C–25°C during day and night, respectively; however, the relative humidity ranged between 60% and 90% during germination period. The seedlings were watered once in 2 days, and their height and stem collar diameter (SCD) were measured at biweekly intervals and continued for 1 year. The seedling height was measured using a 30-cm or 5-m measuring tape and diameter using a digital vernier caliper (150 mm). The mean seedling height and SCD were computed to monthly means. Soil temperature and soil moisture were recorded using a multithermometer and FreshDcart meter, respectively, as mentioned earlier.

### 2.3. Seedling Biomass

The seedling biomass was estimated by harvesting 10 seedlings after 1 year, where the height and diameter of seedlings were measured before cutting or uprooting. The roots were separated from shoots and weighed separately. These samples were dried in an oven (70°C) for 72 h to estimate the dry weight of shoots and roots.

### 2.4. Data Analysis

The data on seed germination and seedling height and diameter were compiled and organized into meaningful tables for analysis. The GP was calculated using Equation (1), mean germination time (MGT) using Equation (2), and germination index and germination energy (GE) using Equations (3) and (4), respectively. Additionally, the mean seedling height and diameter were combined and converted from weeks to months, and the mean monthly increment and monthly relative percentage of height and diameter were calculated. Seedling biomass was calculated following Formulas ((5)–(10)), while Dickson's quality index (DQI) was calculated using Formula (11). One-way ANOVA and post hoc tests (*p* ≤ 0.05) were conducted to compare the germination rate, GE, and other seedling quality parameters. The analyses and correlations were conducted using Statistical Package for Social Sciences (SPSS, Version 22.0), OriginPro 2024b, and Microsoft Excel (Version 2016).

### 2.5. Formula and Equations [15, 36–39]

(1) $$\begin{array}{c} \text{Germination }\left(\%\right)=\frac{\text{germinated seeds}}{\text{total seeds tested}}\times 100, \end{array}$$

(2) $$\begin{array}{c} \text{Mean germination time }\left(\text{MGT}\right)=\frac{\sum T_{i}N_{i}}{S}, \end{array}$$

(3) $$\begin{array}{c} \text{Germination index }\left(\text{GI}\right)=\left(\frac{G1}{1}\right)+\left(\frac{G2}{2}\right)+\cdots +\left(\frac{Gx}{x}\right), \end{array}$$

(4) $$\begin{array}{c} \text{Germination energy }\left(\text{GE}\right)=\text{the percentage of seed germination obtained at maximum daily germination}, \end{array}$$

(5) $$\begin{array}{c} \text{Total fresh biomass }\left(\text{TFB}\right)=\text{fresh root biomass }\left(\text{FRB}\right)+\text{fresh shoot biomass }\left(\text{FSB}\right), \end{array}$$

(6) $$\begin{array}{c} \text{Total dry biomass }\left(\text{TDB}\right)=\text{dry root biomass }\left(\text{DRB}\right)+\text{dry shoot biomass }\left(\text{DSB}\right), \end{array}$$

(7) $$\begin{array}{c} \text{Water content of the root }\left(\text{WCR}\right)=\text{fresh root biomass}-\text{dry root biomass}, \end{array}$$

(8) $$\begin{array}{c} \text{Water content of the shoot }\left(\text{WCS}\right)=\text{fresh shoot biomass}-\text{the dry shoot biomass}, \end{array}$$

(9) $$\begin{array}{c} \text{Total water content }\left(\text{TWC}\right)=\text{WCR}+\text{WCS}, \end{array}$$

(10) $$\begin{array}{c} \text{Total percentage water content }\left(\text{TPWC}\right)=\left(\frac{\text{TWC}}{\text{TFB}}\right)\times 100, \end{array}$$

(11) $$\begin{array}{c} \text{Dickso}{\text{n}}^{\text{'}}\text{s quality index }\left(\text{DQI}\right)=\frac{\text{total dry weight}}{\text{shoot heigt}/\text{stem diamter}+\text{shoot dry weight}/\text{root dry weight}}, \end{array}$$

where *T*_*i*_ is the number of days from beginning of the experiment; *N*_*i*_ is the number of seeds germinated per day; *S* is the total number of seeds germinated; *G* is germination days 1, 2,…; and *x* represent the corresponding day of germination.

## 3. Results

### 3.1. GP, MGT, Germination Index, and GE

**Figure 3:**
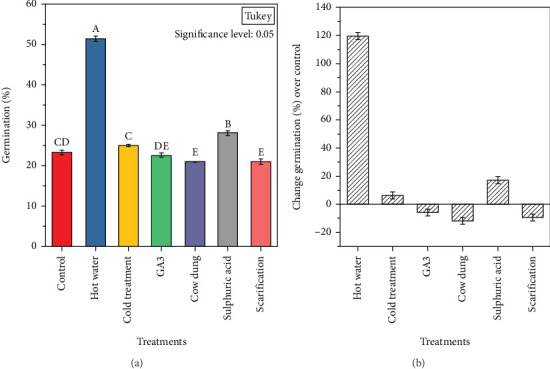
(a) Germination percentage and (b) change germination (%) over control of different treatments of *L. speciosa*. Where the same letters in (a) indicate no significant difference at 0.05 level and different letters indicate a significant difference between treatments.

**Figure 4:**
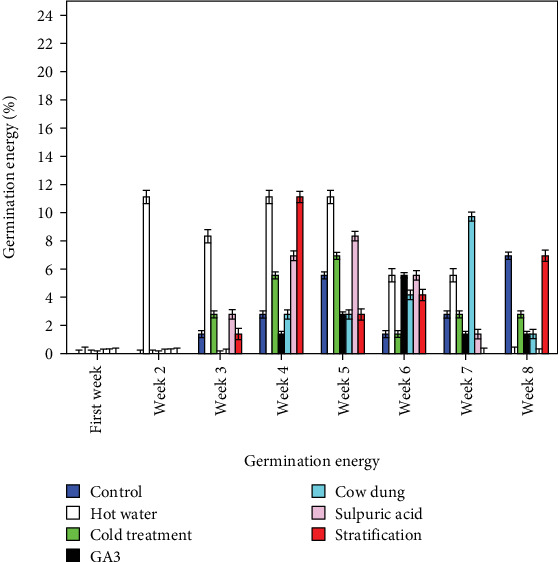
Germination energy of *L. speciosa.*

The seed GP of *L. speciosa* was found to be significantly different between treatments (*p* ≤ 0.05). Among the treatments, hot water recorded the highest GP (51.95%) (Figure 3a). The seeds pretreated in hot water and H_2_SO_4_ showed increased GP, while those treated in cold, GA_3_, cow dung, and scarification showed a decreased GP when compared with the control (Figure 3b). The MGT was minimum in H_2_SO_4_ treatment (21.17 days) and maximum in cold treatment (26.21 day). Conversely, seeds pretreated in H_2_SO_4_ and hot water improved seed germination time by 7.96% and 3.74%, respectively, compared to the control (Table 1). However, the highest germination index was recorded by hot water treatment (0.96), while hot water enhanced seed germination index by 118.18% compared to the control (Table 1). Further, the highest GE was recorded in the second week for the seeds pretreated in hot water (11.11%) (Figure 4). The GE and the number of seeds germinated per day were found to be significantly different (*p* < 0.05) between the treatments.

### 3.2. Seedling Growth Performance

**Figure 5:**
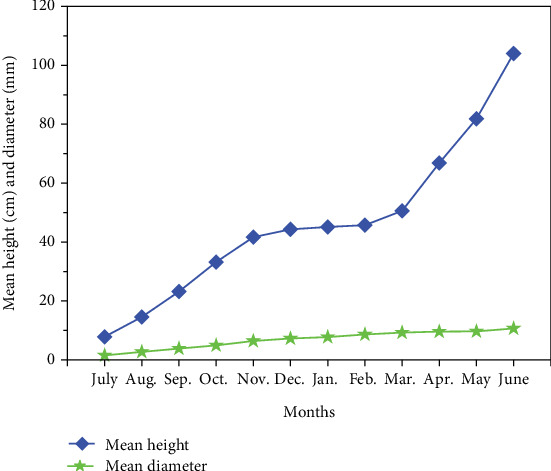
Mean seedling height and diameter of *L. speciosa* per month.

**Figure 6:**
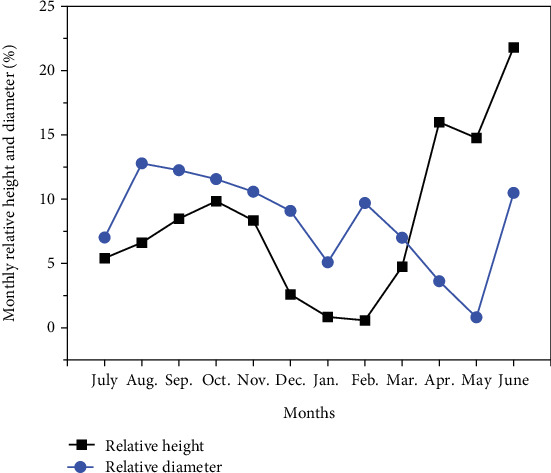
Relative mean seedling height and diameter increment.

After 1 year of seedling growth, the mean seedling height reached up to 104 cm with a diameter of 10.67 mm (Figure 5). The highest mean seedling height increment was recorded in June (22 cm) and diameter in August (1.18 mm) (Table 2), while the increment in seedling height and diameter differed significantly between months (Table 2). The results revealed that the increment in seedling height ceased during the winter months (Figure 6). Moreover, we noticed a strong positive correlation between seedling growth parameters and soil temperature (Table 3).

### 3.3. Seedling Biomass

The seedlings showed 74.86% water content (Table 4). Nevertheless, a strong positive correlation was observed between seedling growth parameters and seedling biomass parameters, while a negative relationship was noticed when these were correlated with the total percentage of water content (Table 5). However, the seedling quality index (DQI) was 2.68 (Table 4). Moreover, there was a strong correlation between DQI with other growth variables, while DQI showed a negative correlation with TPWC, indicating different growth strategies and water use efficiencies (Table 5).

## 4. Discussion

### 4.1. GP, MGT, Germination Index, and GE

Different seed pretreatments such as hot water [12]; scarification of seed coat by nicking, piercing [11], or acid treatment [13]; and other presowing treatments are used to enhance seed germination or defeat physical seed dormancy. However, untreated seeds (usually the drupes) germinate later and unevenly. Azad et al. [11] and Thangjam and Sahoo [15] have highlighted that seeds with a solid, hard inflexible seed coat were documented to enhance germination time and index with presowing treatments. Among the different treatments, soaking in hot water (80°C) for 10 min showed higher seed germination compared to the other treatments. This finding indicates the ability of hot water in breaking the seed coat and in improving seed germination rate more efficiently than other treatments. This argument also finds support from other works [12, 15]. The lowest seed germination recorded by scarification in the present study indicates that some seed parts might have been injured during the operation. However, scarification has been found to be an important pretreatment in improving seed germination rate of some other tree species having hard seed coat [39, 40]. The findings of the present study also revealed significant differences in MGT, starting and closing dates, and germination index among the different treatments. In general, *L. speciosa* seed germination occurs within 2 weeks after sowing. This is fairly similar to another report [19] which mentioned that biochar can enhance the germination of *L. speciosa* and reduce the MGT. In the present study, we observed that MGT was significantly improved after using H_2_SO_4_ thereby reducing it to 7.96% compared to the control and other treatments, while germination index increased by 118.18% compared to the control, showing the role of these treatments in enhancing the germination indices of *L. speciosa*. Furthermore, seeds collected from suitable and healthy mother trees can improve the GP and therefore speed up the plantation programs [41].

Our findings also suggest hot water as the best presowing treatment to improve the seed germination and reduce germination time in *L. speciosa* and for quality seedling production. Although H_2_SO_4_ was reported by several scientists to enhance the seed GP in many tree species, our findings disagree with them. Thangjam and Sahoo [15] reported that the concentration of H_2_SO_4_ may damage the seed embryo when the seeds do not have hard seed coats like *L. speciosa*. Besides, H_2_SO_4_ may not be an easy treatment to look into, as its handling requires some degree of expertise and involvement of associated cost to use it, and therefore, this treatment is not recommended.

### 4.2. Seedling Growth Performance and Biomass

Light and temperature are important prerequisites for seedling growth, and therefore, understanding their role is crucial for predicting regeneration of forest [42]. Our results showed that with the increase in temperature, the seedlings showed better growth increment, a finding that is in agreement with [41, 43, 44]. This is further evident from a strong positive correlation between soil temperature and seedling height growth. During winter months, low temperature caused stunted growth, as there was no increase in growth increments. Similarly, several studies highlighted that seedling/tree growth and allocation pattern might negatively be affected by winter conditions and low soil temperature [45–48]. Likewise, seedlings increased in shoot size with greater exposure to light and favorable temperatures during growing seasons [49, 50]. Moreover, production of quality seedlings is paramount importance in any seed pretreatment studies besides improving seed germination. In the present study, we employed DQI as the tool to evaluate the seedling quality as a function of total dry matter, shoot height, stem base diameter, and shoot dry matter. Our finding suggests that the seedling quality index of *L. speciosa* in the 1-year period is 2.68, which indicates that the seedlings are sufficient uniformity and good quality for transplantation. Many authors have highlighted DQI as a proxy measure to evaluate seedling quality and uniformity to match specific site requirements [40] so as to ensure their survival and better growth [38, 51].

Seedling biomass is yet another important attribute for the evaluation of seedling quality. While interspecific variation in seedling biomass can be critical in the process of population recruitment [52], higher biomass allocation to the root contributes to sturdiness to seedlings and their response to subsequent silvicultural manipulation and/or environment [49, 53]. We found that with the increase of seedling height and diameter, the amount of total water content in seedlings increases, where previous scientists reported that the seedlings grown in shade have a difference in seedling biomass. A study reported that seedlings generally contain a low biomass allocation [54]. Conversely, seedlings grown under higher light availability are generally larger and produce more amount of biomass compared to seedlings subjected to less light [55], although seedling stress rate caused by limited light on seed growth of seedling can depend on species factors including tolerance to shade, competition, plasticity, and availability of other resources [56, 57].

## 5. Conclusion

The findings suggest that hot water is the best pretreatment for ensuring the highest seed germination in *L. speciosa*, while minimum MGT was achieved by H_2_SO_4_ treatment. We also observed that seedling growth parameters were significantly influenced by soil temperature, while a good DQI value (2.68) indicated seedlings of sufficient uniformity and quality during the 1-year period.

## Figures and Tables

**Table 1:** Mean germination time and germination index of *L. speciosa* seeds.

**Table treatment**	**Mean germination time**	**Germination index**
Control	23.00^c^	0.44^c^
Hot water	22.14^e^ (−3.74)	0.96^a^ (+118.18)
Cold treatment	26.21^a^ (+13.96)	0.54^b^ (+22.73)
GA3	24.40^d^ (+6.09)	0.41^c^ (−6.82)
Cow dung	25.00^b^ (+8.70)	0.36^d^ (−18.18)
H_2_SO_4_	21.17^e^ (−7.96)	0.57^b^ (+29.55)
Stratification	23.71^c^ (+3.09)	0.30^d^ (−31.82)

*Note:* The values in parentheses indicate the percentage increase (+)/decrease (−) compared to the control. Treatments sharing the same letter indicate no significant difference at the 0.05 level, while different letters indicate a significant difference between treatments.

**Table 2:** *L. speciosa* monthly seedling height and diameter increment (mean ± SD, *n* = 10).

**Year**	**Month**	**Monthly mean height increment (cm)**	**Monthly mean diameter increment (mm)**
2023	July	—	—
	Aug	6.73 ± 6.46^d^	1.18 ± 1.24^a^
	Sep	8.62 ± 1.18^c^	1.14 ± 0.32^a^
	Oct	10.00 ± 7.22^c^	1.07 ± 0.26^a^
	Nov	8.48 ± 5.48^c^	0.98 ± 0.81^a^
	Dec	2.63 ± 1.03^e^	0.84 ± 0.12^b^

2024	Jan	0.85 ± 0.10^f^	0.47 ± 0.27^d^
	Feb	0.58 ± 0.52^f^	0.90 ± 0.28^a^
	Mar	4.83 ± 4.37^d^	0.65 ± 0.33^c^
	Apr	16.25 ± 10.72^b^	0.33 ± 0.08^d^
	May	15.00 ± 12.76^b^	0.07 ± 0.03^e^
	June	22.17 ± 23.41^a^	0.97 ± 0.69^a^

*Note:* Where the same letter in a column is not significantly different at the 0.05 level.

**Table 3:** Monthly *L. speciosa* seedling characteristics, soil temperature, and soil moisture correlation.

**Variables**	**Mean height (cm)**	**Mean diameter (mm)**	**Mean soil temperature**	**Mean soil moisture**
Mean height (cm)	1			
Mean diameter (mm)	0.91⁣^∗∗^	1		
Mean soil temperature	0.56	0.40	1	
Mean soil moisture	−0.20	−0.07	0.02	1

⁣^∗^Correlation is significant at the 0.05 level (2-tailed).

⁣^∗∗^Correlation is significant at the 0.01 level (2-tailed).

**Table 4:** Mean biomass of *L. speciosa* seedlings and Dickson's quality index (DQI) (mean ± SD, *n* = 10).

**Parameters**	**M** **e** **a** **n** ± **SD**
Mean height (cm)	108.67 ± 28.62
Mean SCD (mm)	10.15 ± 3.72
Mean TFB (g)	142.03 ± 16.36
Mean TDB (g)	38.06 ± 46.19
Mean TWC (%)	103.97 ± 11.22
Mean TPWC (%)	74.86 ± 2.73
Mean DQI	2.68 ± 2.89

Abbreviation: SD, standard deviation.

**Table 5:** *L. speciosa* seedling characteristic correlation with biomass variables.

**Variables**	**Height (cm)**	**Diameter (mm)**	**TFB**	**TDB**	**TWC**	**TPWC**	**DQI**
Height (cm)	1						
Diameter (mm)	0.95	1					
TFB	0.97	0.99⁣^∗^	1				
TDB	0.96	0.99⁣^∗^	0.99⁣^∗^	1			
TWC	0.97	0.99	0.99⁣^∗∗^	0.99⁣^∗^	1		
TPWC	−0.63	−0.85	−0.80	−0.83	−0.79	1	
DQI	0.94	0.98	0.98	0.98	0.98	−0.86	1

⁣^∗^Correlation is significant at the 0.05 level (2-tailed).

⁣^∗∗^Correlation is significant at the 0.01 level (2-tailed).

## Data Availability

All data used/analysed in this paper are available with the corresponding author and will be shared on a reasonable request.
